# Sex allocation promotes the stable co-occurrence of competitive species

**DOI:** 10.1038/srep43966

**Published:** 2017-03-06

**Authors:** Kazuya Kobayashi

**Affiliations:** 1 Laboratory of Insect Ecology, Graduate School of Agriculture, Kyoto University, Kyoto 606-8502, Japan

## Abstract

Biodiversity has long been a source of wonder and scientific curiosity. Theoretically, the co-occurrence of competitive species requires niche differentiation, and such differences are well known; however, the neutral theory, which assumes the equivalence of all individuals regardless of the species in a biological community, has successfully recreated observed patterns of biodiversity. In this research, the evolution of sex allocation is demonstrated to be the key to resolving why the neutral theory works well, despite the observed species differences. The sex allocation theory predicts that female-biased allocation evolves in species in declining density and that this allocation improves population growth, which should lead to an increase in density. In contrast, when the density increases, a less biased allocation evolves, which reduces the population growth rate and leads to decreased density. Thus, sex allocation provides a buffer against species differences in population growth. A model incorporating this mechanism demonstrates that hundreds of species can co-occur over 10,000 generations, even in homogeneous environments, and reproduces the observed patterns of biodiversity. This study reveals the importance of evolutionary processes within species for the sustainability of biodiversity. Integrating the entire biological process, from genes to community, will open a new era of ecology.

Biodiversity has long been a source of wonder and scientific curiosity^1^. An important measure of biodiversity at a specific trophic level is the species abundance distribution, which is usually illustrated in a rank-abundance diagram (RAD), where the logarithm of abundance is plotted against the rank of species in abundance^2^,^3^,^4^. RADs show a certain pattern over a wide range of ecological communities, which suggests that a general theory of biodiversity should apply. Two major groups of theoretical models have been designed to explain this pattern^5^,^6^,^7^,^8^. One group of models is based on niche theory, where each species occupies a niche in a multi-dimensional resource space specifically used by the focal species. Diversity is determined primarily by the number of available niche spaces; thus, this theory predicts that within-species differences in niche availability are a key feature of high species diversity. The other group of models is based on the neutral theory, which assumes that all individuals have the same demographic parameters, regardless of their species identity. Although this assumption appears to be unrealistic, the neutral theory has successfully recreated the RADs observed in nature^2^,^9^. which has generated a vigorous debate among ecologists because the success of the neutral theory appears paradoxical in light of the manifest differences among species.

The study of sex allocation is one of the most productive fields in evolutionary biology^10^,^11^,^12^,^13^,^14^,^15^,^16^,^17^,^18^, with a large empirical literature to support the theoretical predictions; however, sex allocation is a frequently overlooked feature of sexual organisms in community ecology research. Sex allocation is strictly linked to the population dynamics of a species because allocations to male reproductive functions (such as pollen or male offspring) do not directly contribute to population growth. Generally, the sex allocation theory predicts equal allocation to male and female reproductive functions for the optimal division of resources^10^,^11^. However, such allocations are based on certain assumptions, such as random mating and a large mating population. In plant species with low density (i.e., a few individuals in the pollen dispersal area), allocation to pollen reduces the mating success rates for each pollen grain due to the limitation of available mating partners, whereas greater allocation to seeds creates more mating partners for pollen grains. Thus, this intraspecific competition over mating promotes the evolution of excess female-biased allocation at a low density. For example, if only one individual occurs in the pollen dispersal area, then this individual should allocate most of its resources to seeds because a sufficient amount of pollen is available to fertilize all of the seeds. Hamilton^12^ developed a generalized model that includes low and high density conditions. In this model, an evolutionarily stable allocation to females (seeds) is (*n* + 1)/2*n*, where *n* represents the number of individuals in a local mating population (pollen dispersal area). If *n* is sufficiently large, equal allocation (0.5) occurs. In community ecology, the most important aspect of this formula is that adaptive allocation depends on the density of the species or on the number of conspecific individuals in the pollen dispersal area. Therefore, the theory predicts that female-biased allocation evolves in species with declining density and that this allocation improves population growth, which should lead to an increase in the density. In contrast, when the density increases, a less biased allocation evolves, which reduces the population growth rate and leads to decreased density. Previous studies^19^,^20^,^21^ have shown that this negative density-dependent effect of adaptive sex allocation on population growth allows two competitive species to co-occur in a homogeneous environment that does not allow niche differentiation. In this study, the previous model^21^ was generalized and applied to multiple species to show that the co-occurrence of sexual organisms persists without niche differentiation. Moreover, it is demonstrated that the model is capable of recreating the S-shaped form of a RAD that is observed in nature.

## Model

To show the effect of sex allocation on an ecological community, an individual-based model was constructed that assumes a community of haploid annual plant species in a homogeneous environment. The spatial structure of the model followed an island model in which individuals belonged to one of the local populations that corresponded to the pollen dispersal area. In the model, each individual allocates a species-specific amount of resources to pollen and seeds depending on the individuals’ genetic value of locus *g* (0 < *g* < 1). This species-specific amount of available resources reflects differences in the species-level adaptation to the environment, e.g., photosynthetic capabilities under certain light conditions. Each seed is fertilized by a single pollen grain that is randomly selected from the pool of total pollen produced by conspecific individuals within the pollen dispersal area. To simplify the model, self-compatible species were assumed, which ensured fertilization for any seed. Individuals in the next generation were randomly selected from the pool of total seeds produced by the entire community; these individuals inherit genes randomly from either the mother or father. The total number of individuals was fixed over the generations. Random mutation occurred with a constant probability per generation per locus (i.e., 0.001); then, gene (*g*) was replaced with a uniform random number between 0 and 1. All of the seeds that were not selected as individuals in the next generation die, which assumes that a seed bank is not available in the soil. After the blooming season, all of the flowered individuals die, and the next generation starts. The simulations began with individuals that presented random genetic values (a uniform distribution between 0 and 1), and the species of each individual were randomly decided with equal probability. The simulation code can be accessed as a supplementary file.

## Results and Discussion

**Figure 1:**
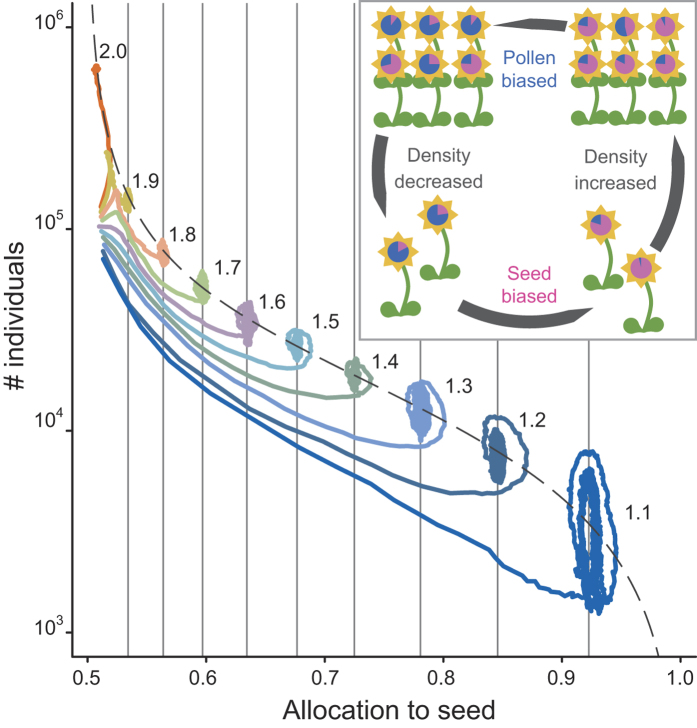
Simulation results showing the evolutionary dynamics of sex allocation and demographics over 2,000 generations. Each coloured line corresponds to the dynamics of each species and is a plot of the logarithm of species abundance against the mean allocation to female reproductive function (seeds), starting from approximately 10^5^ individuals with equal allocation (0.5). The number on each coloured line indicates the amount of available resources for the species. The dashed line is the theoretical prediction: *s* = (*λ* + 1 − exp(−*λ*))/2*λ*, where *s* and *λ* indicate an evolutionarily stable allocation to females and the expected number of conspecific individuals in the pollen dispersal area (local population), respectively. The grey vertical lines indicate allocations in which the population growth rate of a certain species is equal to that of the dominant species with allocation observed at the end of the simulation. The total number of individuals and the number of local populations in the community are 10^6^ and 10^4^, respectively. Thus, each local population contains 100 individuals, and the density *λ* multiplied by the number of local populations 10^4^ becomes the total number of individuals for each species (y-axis). The inset panel illustrates how the evolution of sex allocation stabilizes the density of each species. The circles in the flowers reflect the individuals’ allocations to seeds (pink) and pollen (blue).

In the model, species-specific amounts of resources were sequentially set from 2 to 1.1 at an equal interval (0.1), and the results indicate that 10 species co-occurred over 2,000 generations (Fig. 1). Although the dominant species presents the largest quantity of available resources in the community, less dominant species persisted because the high-density species allocate approximately half of their resource to male functions (pollen) due to intraspecific competition over mating, which reduces their population growth rates (production of seeds). Similarly, less dominant species cannot easily increase their densities because increased density improves the genetic contribution of pollen to the next generation within the species, which inhibits the evolution of an extremely female-biased allocation. The evolution of sex allocation induces invariant population growth rates among species that share an environment and receive unequal amounts of resources; therefore, sex allocation leads to the stable co-occurrence of multiple species, even in homogeneous environments. The model presented here predicts cyclic dynamics in demographics and sex allocations, and that the sex allocations of rare species are more female-biased than those of common species because they evolve depending on the species density (Fig. 1).

**Figure 2:**
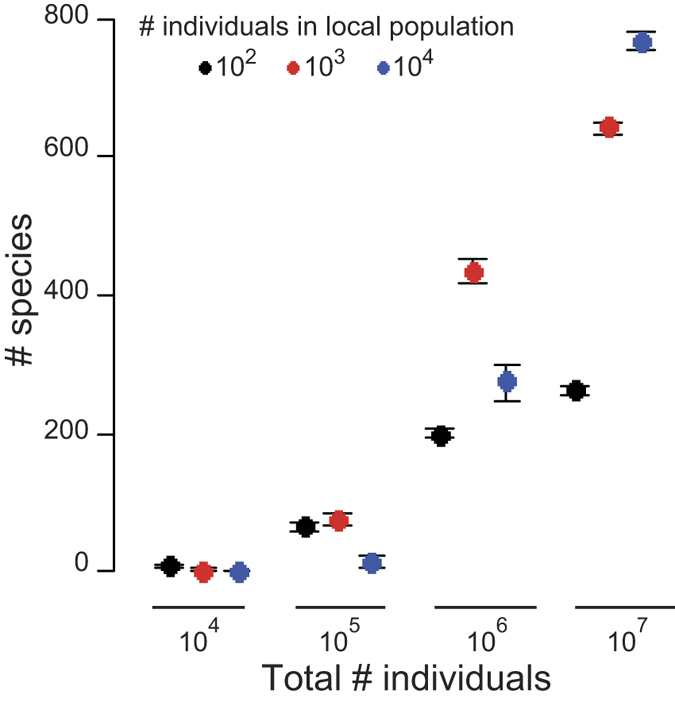
The large space improves the persistence of a considerable number of species over 10,000 generations. The mean number of species co-occurring at the end of the simulations (10,000 generation) are plotted against the spatial scale (total number of individuals in the community), with error bars representing one standard deviation. In each plot, 100 simulations were performed. The species-specific amounts of available resources were sequentially set from 2 to 1.001 at equal intervals.

The model also indicates that the species with relatively few available resources generate a small population size and occasionally go extinct. This extinction arises not only from interspecific competition over space but also from their small population size, which reduces genetic variation and the possibility of new mutations, both of which are essential for the evolution of female-biased allocation. Thus, these species stochastically fail to achieve female-biased allocations before extinction due to their small population size. In other words, under these simulated condition, extinction should never occur when there is infinite space. Indeed, in a simulation that began with 1,000 species and when species-specific amounts of available resources are sequentially set from 2 to 1.001 at an equal interval, the number of species co-occurring over 10,000 generations was positively correlated with the total number of individuals in the community (Fig. 2). This result suggests that with infinite space, any species can survive, even if it does so at an infinitely small frequency, despite the homogeneity of the environment and the differences in the amount of resources allocated by each species to reproductive functions.

**Figure 3:**
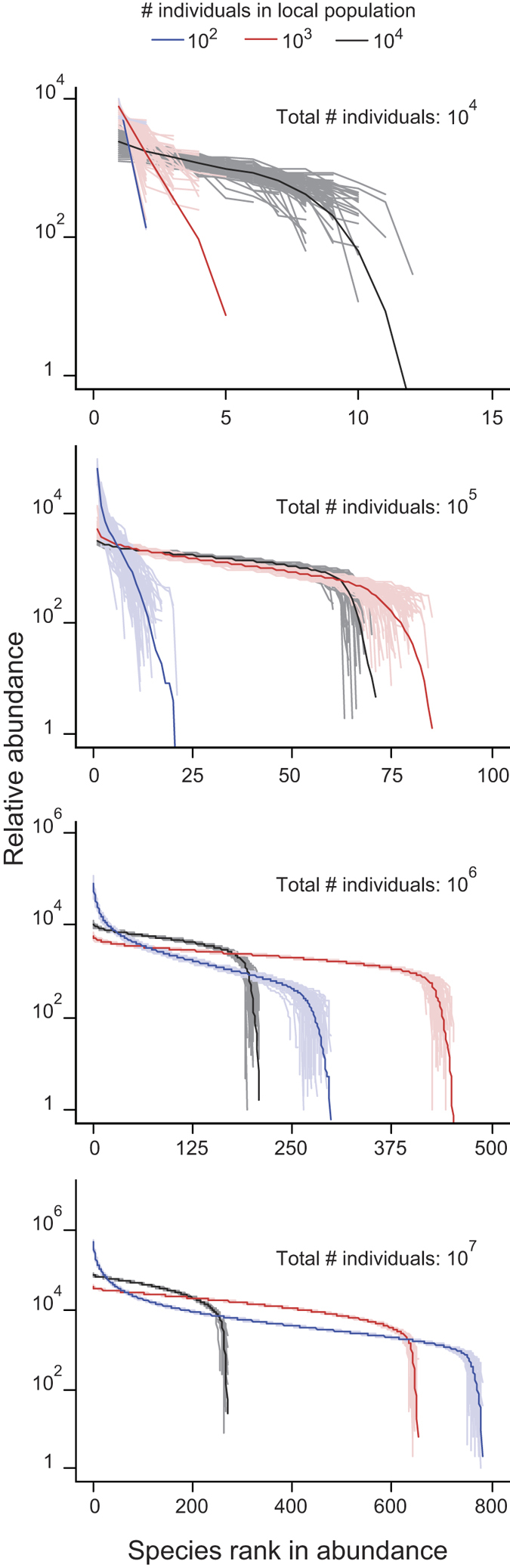
Species rank abundance diagrams drawn from the results of Fig. 2. The light and dark coloured lines correspond to the results of a single simulation run and the mean of 100 simulation runs, respectively.

At the end of the simulations, the RADs exhibited an S-shaped form (Fig. 3), which is common for field data^2^,^3^,^4^. This result is not surprising because the evolutionary dynamics, including interspecific competition over space and intraspecific competition over mating, produce RADs in this form. To explain this phenomenon, a modification was required in the equation of Hamilton’s sex allocation theory^12^, as follows:


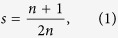


where *s* and *n* represent an evolutionarily stable allocation to females (seeds) and the number of individuals in a local population, respectively. In Hamilton’s formula, the number of individuals of the focal species is fixed in any local population. In the model presented here, rare species are absent in certain local populations and sex allocation does not occur in these local populations. Therefore, *n* should be replaced by the expectation of a zero-truncated Poisson distribution:


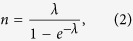


where *λ* indicates the density of the species, i.e., the expected number of conspecific individuals in the pollen dispersal area (local population). Thus, an evolutionarily stable sex allocation can be written as a function of the density as follows:


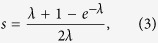


In regions under and above this equation (the dashed line in Fig. 1), female- and male-biased allocation evolves in the species, respectively. Similarly, whether the population size of a species increases or decreases is switched with the threshold line (the grey vertical lines in Fig. 1) on which the population growth rate of the species is consistent with that of other species in the community. Therefore, this evolutionary dynamics maintains the sex allocation and density of any species close to the line of this equation (Figs 1 and 3). For species that exhibit densities close to this line, the RADs inevitably form an S-shape.

**Figure 4:**
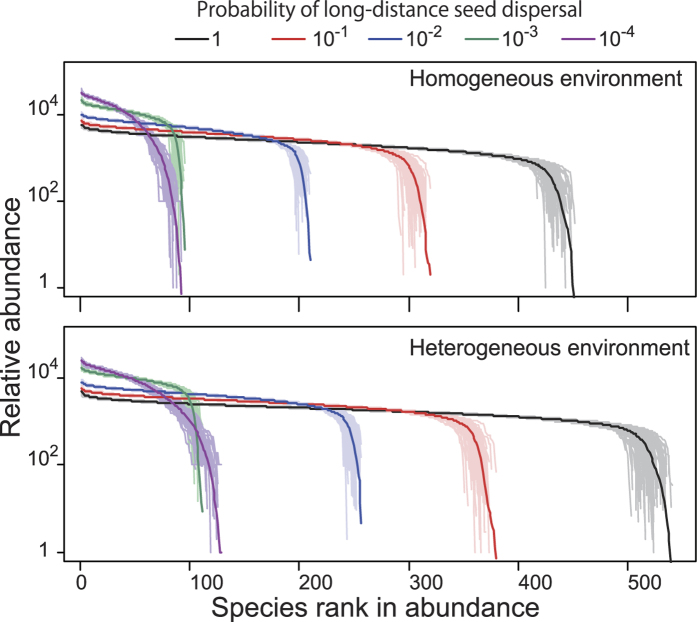
Species rank abundance diagrams for the simulation runs after 10,000 generations. The light and dark coloured lines represent the results of the single simulation run and the mean of 100 simulation runs, respectively. For all of the simulations, the total number of individuals in the community is 10^6^. The pollen and seed dispersal areas contain 10^3^ and 10^2^ individuals, respectively.

To simulate more realistic conditions, two additional factors were incorporated into the model. One factor was a limitation of the seed dispersal range. In the above models, the individuals of the next generation were randomly chosen from the seeds produced in the entire community, which is unlikely to occur in nature because the dispersal distance of seeds is generally shorter than that of pollen. Therefore, the model was modified as follows: the probability of randomly selecting individuals of the next generation from seeds produced throughout the entire community (long-distance dispersal) was small (*d*), and the remaining individuals are selected from seeds produced in the local area (short-distance dispersal), which is smaller than the pollen dispersal area. The forms of RADs are robust with this modification (Fig. 4). The other factor incorporated into the model is a heterogeneous environment that permits niche differentiations among species. Here, two types of environment were incorporated into the model. The first type of environment was the same as in the above model, where the species-specific amount of available resources was sequentially set from 2 to 1.001 at equal intervals and occupied three-quarters of the region in the model. The residual region represented the other type of environment, where the species-specific amount of available resources was set as a reverse sequence from 1.001 to 2 at equal intervals. This heterogeneous environment increased the number of persistent species compared with that of a homogeneous environment, although the RAD forms were maintained (Fig. 4). Note that the qualitatively same results were obtained from the simulations with three different conditions: lower mutation rates (Supplementary Fig. S1), another pattern of mutation that is perturbing existing genetic values (Fig. S2), and a normal distribution for the species-specific amount of resources (Fig. S3). These results demonstrate that sex allocation provides a buffer against species differences regardless of niche availability and causes invariance in population growth rates among the species. Therefore, the model consistently reproduced the S-shaped form of RADs, even after these modifications.

To focus on how the S-shaped form of RADs arises from the negative density-dependent effect of intraspecific competition over mating, simple conditions were assumed, and thus many interesting topics remain uncovered. For example, the model supposed no inbreeding depressions and complete self-compatibility for any species, which ensures the mating success of any seeds via selfing. These factors will strongly influence population dynamics, especially when population size decreases rapidly. If the species density decreases gradually, it would be expected that most deleterious genes are purged out and self-compatibility would evolve, which would allow the species long-term continuation with excess seed-biased allocations at low density. Interestingly, the model presented here predicted that such rare species have the potential to be invasive alien species when they obtain large amounts of resources in introduced environments due to their self-compatibility, lack of inbreeding depressions and excess seed-biased allocations. After a rapid increase in density of the invaded species, the evolution of sex allocation toward equal numbers in the invaded species gradually reduces its impact on other native species. During this invasion process, some native species in the invaded region will go extinct when the evolution of female-biased allocation is delayed. Therefore, integrating the effects of inbreeding depressions and self-compatibility into the model is an important direction not only for community ecology but also for conservation biology.

Although the neutral theory does not consider species differences, it successfully recreates general species abundance patterns^2^,^9^. However, in the real world, species are different from each other. The model presented here demonstrates how the evolution of sex allocation mitigates species differences in population growth rates. Thus, the sex allocation theory has the potential to reconcile the niche theory and the neutral theory. Considering the dominance of sexual reproduction in nature, sex allocation must be ubiquitous. Although allocating resources to male reproductive functions does not appear to be a strategy for increasing population growth, commonly observed species allocate approximately half of their resources to males^10^,^11^,^12^. Together with previous findings, this study provides insights into the mystery of biodiversity and shows how the intrinsic feature of sexual organisms permits the stable co-occurrence of competitors over a broad range of environments.

## Additional Information

**How to cite this article:** Kobayashi, K. Sex allocation promotes the stable co-occurrence of competitive species. *Sci. Rep.* **7**, 43966; doi: 10.1038/srep43966 (2017).

**Publisher's note:** Springer Nature remains neutral with regard to jurisdictional claims in published maps and institutional affiliations.

## Supplementary Material

Supporting Information
