# Supplementary material for: Sex allocation promotes the stable co-occurrence of competitive species
Source: Sci Rep. 2017 Mar 6;7:43966. doi: 10.1038/srep43966 (PMC5338262; doi:10.1038/srep43966)
Supplement: Supporting Information [file srep43966-s1.doc]

**Supporting Information file**

**Title:** Sex allocations promotes the stable co-occurrence of competitive species

**Authors:** Kazuya Kobayashi^*^

**Affiliations:**

Laboratory of Insect Ecology, Graduate School of Agriculture, Kyoto University, Oiwake-cho, Kitashirakawa, Sakyo-ku, Kyoto 606-8502, Japan

* Correspondence to: kobakaz@kais.kyoto-u.ac.jp

**
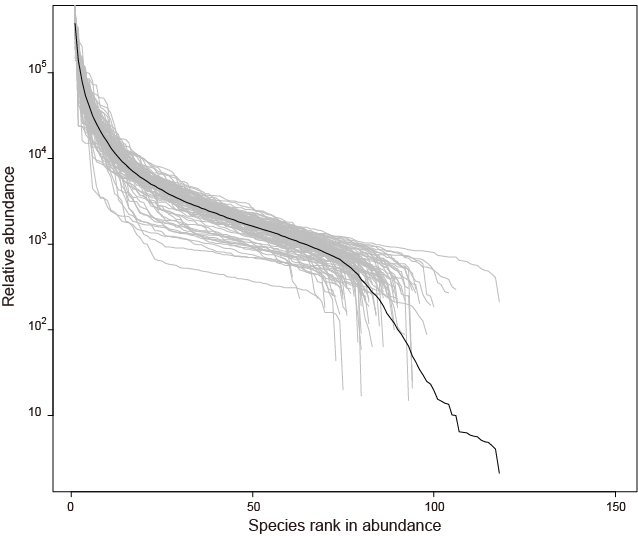
**

**Figure S1.** Species rank abundance diagrams drawn from the simulation results supposing lower mutation rates (10^-5^).

The light and dark coloured lines correspond to the results of a single simulation run and the mean of 100 simulation runs, respectively. The total number of individuals and local populations (pollen dispersal areas) in the community is 10^6^ and 10^3^, respectively. Thus, the pollen dispersal areas contain 10^3^ individuals.


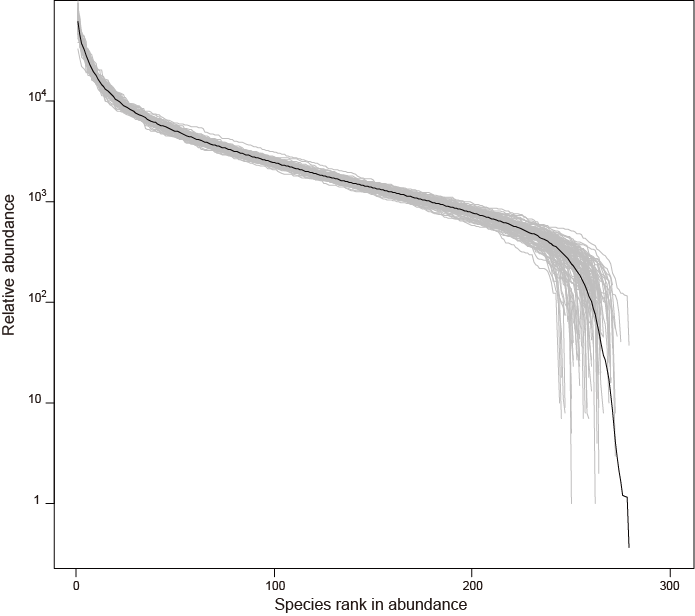


**Figure S2.** Species rank abundance diagrams drawn from the simulation results supposing another pattern of mutation.

In this simulations, genetic value of the mutant is the mean of existing genetic value and a uniform random value between 0 and 1. Light and dark coloured lines correspond to the results of a single simulation run and the mean of 100 simulation runs, respectively. The total number of individuals and local populations (pollen dispersal areas) in the community is 10^6^ and 10^3^, respectively. Thus, the pollen dispersal areas contain 10^3^ individuals.

**
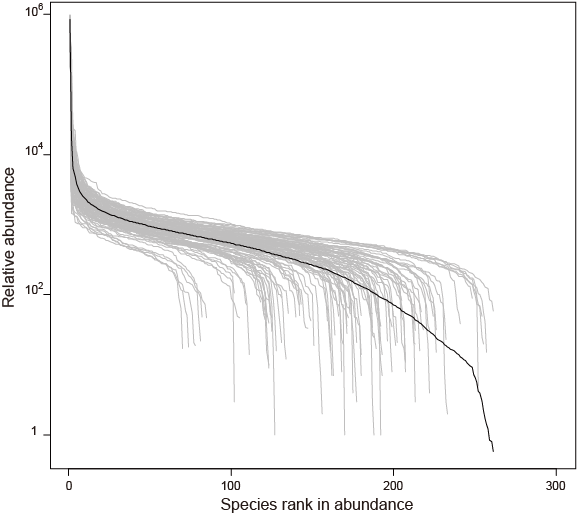
**

**Figure S3.** Species rank abundance diagrams drawn from the simulation results using a normal distribution for the species-specific amount of resources (mean = 1, sd = 0.1).

Light and dark coloured lines correspond to the results of a single simulation run and the mean of 100 simulation runs, respectively. The total number of individuals and local populations (pollen dispersal areas) in the community is 10^6^ and 10^3^, respectively. Thus, the pollen dispersal areas contain 10^3^ individuals.

// Simulation source code for Linux icpc

//

//#include "stdafx.h"

#include <boost/random.hpp>

#include <random>

#include <iostream>

#include <fstream>

#include <sstream>

#include <string>

#include <time.h>

#include <vector>

#include <numeric>

#include <algorithm>

using namespace std;

int main(int argc,char *argv[])

{

int i, j, k;

cout << "int argv 1: " << argv[1] << endl;

//Read parameters from txt file

double Parameters[10] = {};

ifstream inputfile("Parameters.txt", ios::in); //read only

string parameter_name;

if (inputfile.fail()){

cout << "Cannot find parameter file(Parameters.txt). press Enter key." << endl;

getchar();

return -1;

}

i = 0;

while (true){//

if (i % 2 == 0){

inputfile >> parameter_name;

if (inputfile.fail()) break;

cout << parameter_name << " = ";

}

else {

inputfile >> Parameters[i / 2];

if (inputfile.fail()) break;

cout << Parameters[i / 2] << endl;

}

i++;

}

inputfile.close();

cout << "parameter read end" << endl;

/* An example of "Parameters.txt"

WholePopulationSize 1000000

PopulationSizeForPollen 10000

PopulationSizeForSeed 1000000

Num_species 1000

MutationRate 0.001

DeathRate 1.0

DispersalRate 1.0

TimeSteps 10000

Niche 0

NicheDensity 1.0

*/

//Setting

int WholePopulationSize = int(Parameters[0]);

int PopulationSizeForPollen = int(Parameters[1]);

int PopulationSizeForSeed = int(Parameters[2]);

int Num_species = int(Parameters[3]); //usually 1000 except for Fig.1

double MutationRate = Parameters[4]; //usually 0.001 except for Fig.S1

double DeathRate = Parameters[5]; //fixed 1.0 (only annual species supposed)

double DispersalRate = Parameters[6]; //usually 1.0 except for Fig.4 (the probability of long-distance dispersal)

int TimeSteps = int(Parameters[7]); //fixed 10000

int TFNiche = int(Parameters[8]); //0 and 1 means homogeneous and homogeneous environment respectively

double NicheDensity = double(Parameters[9]); //functional only when TFNiche is 1, amount of available resource is reversed.

if (WholePopulationSize % PopulationSizeForPollen != 0){

cout << "WholePopulationSize:" << WholePopulationSize << endl;

cout << "PopulationSizeForPollen:" << PopulationSizeForPollen << endl;

cout << "WholePopulationSize is indivisible by PopulationSizeForPollen. press Enter key." << endl;

getchar();

return -1;

}

if (WholePopulationSize % PopulationSizeForSeed != 0){

cout << "WholePopulationSize:" << WholePopulationSize << endl;

cout << "PopulationSizeForSeed:" << PopulationSizeForSeed << endl;

cout << "WholePopulationSize is indivisible by PopulationSizeForSeed. press Enter key." << endl;

getchar();

return -1;

}

int Num_MatingPopulation = WholePopulationSize / PopulationSizeForPollen;

//allocate memory

vector<unsigned short> Species(WholePopulationSize, 0); //Species

vector<double> AllocationToSeed(WholePopulationSize, -1); //AllocationToSeed (0-1; Allocation to female function)

vector<unsigned short> N_Species(WholePopulationSize, 0); //Species for Next population

vector<double> N_AllocationToSeed(WholePopulationSize, -1); //AllocationToSeed for Next population

vector<vector<double>> Pollen(Num_MatingPopulation, vector<double>(Num_species, 0)); //amount of pollen of each species at each subpopulation

vector<double> PollenCounter(WholePopulationSize, 0); //amount of pollen of each species

vector<double> SeedCounter(WholePopulationSize, 0); //amount of produced seeds

vector<unsigned short> Niche(WholePopulationSize, 0); //Niche type

//Random function

random_device rd; // non-deterministic generator

mt19937 gen(rd()); // to seed mersenne twister.

uniform_int_distribution<int> sp(0, (Num_species - 1)); //decide species

uniform_int_distribution<int> rbinom(0, 1); //random binomial

uniform_real_distribution<double> runif(0.0, 1.0); //random uniform

normal_distribution<double> SSAR(1.0, 0.1); //normal distribution for species-specific amount of resources for Fig.S3

vector<double> Productivity(Num_species, 0); //relative productivity for each species (from 2 to 1)

double dif = 1 / double(Num_species);

for (i = 0; i < Num_species; ++i){

Productivity[i] = 2 - i * dif; //for uniform distribution.

//Productivity[i] = SSAR(gen); //for normal ditribution. For Fig.S3.

}

dif += 3.0;//for hetergeneous environments

if(TFNiche == 1){ //Create heterogeneous enviroment or use default homogeneous environement

for (i = 0; i < WholePopulationSize; ++i){

if(runif(gen) < NicheDensity){

Niche[i] = 1;

}

}

}

//allocate memory for output

vector<vector<double>> AllocationToSeed_sp(TimeSteps, vector<double>(Num_species, 0)); //output of each end of timestep

vector<vector<double>> Number_sp(TimeSteps, vector<double>(Num_species, 0));

time_t t = time(NULL);

const tm* lt = localtime(&t);

char time_str[81];

strftime(time_str,sizeof(time_str),"%Y%m%d_%H%I%S", lt);

cout << "repeat #" << argv[1] << " start at " << time_str << endl;

//initialize population

for (i = 0; i < WholePopulationSize; ++i){

Species[i] = sp(gen);

AllocationToSeed[i] = runif(gen);

}

for (int t = 0; t < TimeSteps; ++t) {

//cout << "-----------------------------Timestep start----------------------------" << endl;

//cout << "Generation:" << t << endl;

//cout << "------------------------------Pollination------------------------------" << endl;

for (i = 0; i < WholePopulationSize; ++i){//reset

int subpop = i / PopulationSizeForPollen;

Pollen[subpop][Species[i]] = 0;

}

for (i = 0; i < WholePopulationSize; ++i){

int subpop = i / PopulationSizeForPollen;

if(Niche[i] == 1){

Pollen[subpop][Species[i]] += (1 - AllocationToSeed[i])*(dif - Productivity[Species[0]]); //total pollen at the i-th subpopulation for each species

} else {

Pollen[subpop][Species[i]] += (1 - AllocationToSeed[i])*Productivity[Species[0]]; //total pollen at the i-th subpopulation for each species

}

PollenCounter[i] = Pollen[subpop][Species[i]];

}

//cout << "-------------------------------Death&Birth-------------------------------" << endl;

if(Niche[0] == 1){ // this is always false for homogeneous environment

SeedCounter[0] = AllocationToSeed[0] * (dif - Productivity[Species[0]]);

} else {

SeedCounter[0] = AllocationToSeed[0] * Productivity[Species[0]];

}

for (i = 1; i < WholePopulationSize; ++i){

if(Niche[i] == 1){

SeedCounter[i] = AllocationToSeed[i] * (dif - Productivity[Species[i]]) + SeedCounter[i - 1];

}else{

SeedCounter[i] = AllocationToSeed[i] * Productivity[Species[i]] + SeedCounter[i - 1];

}

}

for (i = 0; i < WholePopulationSize; ++i){

//decide the mother of seed for each site

if (DeathRate > runif(gen)){ //the site occupied by the new individual

if (DispersalRate > runif(gen)){ //the site occupied by the seed randomly chosen from the whole population

double random_choice = runif(gen);

double SeedThreshold = random_choice * SeedCounter[WholePopulationSize - 1];

int expected_individual = int(random_choice * WholePopulationSize);

if (SeedThreshold > SeedCounter[expected_individual]){

for (j = expected_individual + 1; j < WholePopulationSize; ++j){

if (SeedThreshold < SeedCounter[j]){

break;

}

}

}

else{

for (j = expected_individual; j >= 0; --j){

if (SeedThreshold >= SeedCounter[j]){

++j;

break;

}

}

}

if (j < 0){ j = 0; }

} else { //the site occupied by the seed randomly chosen from the local population

int focused_subpop = i / PopulationSizeForSeed;

int start_id = focused_subpop * PopulationSizeForSeed;

int end_id = start_id + PopulationSizeForSeed ;

double random_choice = runif(gen);

double SeedThreshold = 0.0;

if (start_id == 0){

SeedThreshold = random_choice * SeedCounter[end_id - 1];

}else{

SeedThreshold = random_choice * (SeedCounter[end_id - 1] - SeedCounter[start_id - 1]) + SeedCounter[start_id - 1];

}

for (j = start_id; j < end_id; ++j){

if (SeedThreshold < SeedCounter[j]){

break;

}

}

}

// the seed of the individual "j" gets the site

int Reproducing_species = Species[j];

N_Species[i] = Reproducing_species;

///* Random mutation model

if (MutationRate > runif(gen)){

N_AllocationToSeed[i] = runif(gen);

}

else if (rbinom(gen)){

//cout << "inherit from mother" <<endl;

N_AllocationToSeed[i] = AllocationToSeed[j];

}

else {

//cout << "inherit from father " << Reproducing_species << " " << j << endl;

int focused_subpop = j / PopulationSizeForPollen;

double PollenThreshold = runif(gen) * Pollen[focused_subpop][Reproducing_species];

int start_id = focused_subpop * PopulationSizeForPollen;

int end_id = start_id + PopulationSizeForPollen;

for (k = start_id; k < end_id; ++k){

if (Reproducing_species == Species[k]){ //

if (PollenThreshold < PollenCounter[k]){

N_AllocationToSeed[i] = AllocationToSeed[k];

break;

}

}

}

}

//*/

/* perturbing mutation model. For Fig.S2.

if (rbinom(gen)){

//cout << "inherit from mother" <<endl;

N_AllocationToSeed[i] = AllocationToSeed[j];

}

else {

//cout << "inherit from father " << Reproducing_species << " " << j << endl;

int focused_subpop = j / PopulationSizeForPollen;

double PollenThreshold = runif(gen) * Pollen[focused_subpop][Reproducing_species];

int start_id = focused_subpop * PopulationSizeForPollen;

int end_id = start_id + PopulationSizeForPollen;

for (k = start_id; k < end_id; ++k){

if (Reproducing_species == Species[k]){ //

if (PollenThreshold < PollenCounter[k]){

N_AllocationToSeed[i] = AllocationToSeed[k];

break;

}

}

}

}

if (MutationRate > runif(gen)){ //mutation occurs

N_AllocationToSeed[i] = (N_AllocationToSeed[i] + runif(gen) )/2; //replaced by mean

}

//*/

}

else{ //The individual survives to next generation

N_Species[i] = Species[i];

N_AllocationToSeed[i] = AllocationToSeed[i];

}

}

//cout << "----------------------------Replace&Observe------------------------------" << endl;

for (i = 0; i < WholePopulationSize; ++i){

Species[i] = N_Species[i];

AllocationToSeed[i] = N_AllocationToSeed[i];

AllocationToSeed_sp[t][Species[i]] += AllocationToSeed[i];

++Number_sp[t][Species[i]];

}

for (i = 0; i < Num_species; ++i){

AllocationToSeed_sp[t][i] /= double(Number_sp[t][i]);

}

}//end of time steps

//cout << "----------------------------------Output---------------------------------" << endl;

//cout << tempDay[6] << tempDay[7] << tempDay[0] << tempDay[1] <<tempDay[3] << tempDay[4] << endl;

ostringstream Filepath1;

Filepath1 << time_str << "SP" << Num_species << "FPS" << PopulationSizeForSeed << "MPS" << PopulationSizeForPollen << "DR" << DispersalRate << "Rep" << argv[1] << ".csv";

ofstream ofs1(Filepath1.str());

for (int x = 0; x < TimeSteps; ++x){

for (i = 0; i < Num_species; ++i){

ofs1 << AllocationToSeed_sp[x][i] << ",";

}

ofs1 << ",";

for (i = 0; i < Num_species; ++i){

ofs1 << Number_sp[x][i] << ",";

}

ofs1 << endl;

}

return 0;

}
